# The Liver as Another Possible Target Organ for* Bacillus cereus* Infection

**DOI:** 10.1155/2016/7438972

**Published:** 2016-11-29

**Authors:** Mario Alessandri-Bonetti, Umberto Vespasiani-Gentilucci, Giacomo Luppi, Silvia Angeletti, Giordano Dicuonzo, Antonio Picardi

**Affiliations:** ^1^Internal Medicine and Hepatology, Campus Bio-Medico University of Rome, Rome, Italy; ^2^Department of Interventional Radiology, Campus Bio-Medico University of Rome, Rome, Italy; ^3^Clinical Pathology and Microbiology Laboratory, Campus Bio-Medico University of Rome, Rome, Italy

## Abstract

A case of liver abscess due to* Bacillus cereus* infection in an immunocompetent 59-year-old man is reported. Percutaneous drainage and antimicrobial therapy, with vancomycin and levofloxacin afterwards, have been demonstrated to be an appropriate treatment, leading to clinical and radiological cure.

## 1. Introduction

*Bacillus cereus* (c) is an aerobic or facultative anaerobic Gram-positive, spore-forming bacterium. Its natural reservoir includes soil, decaying organic matter, marine water, intestinal tract of invertebrates, vegetables, and other common foods, and the spore is refractory to extreme environmental conditions such as alcohol-based hand-washing products, pasteurization, or *γ*-radiations [1]. Bc group strains manifest a relevant heterogeneity concerning lifestyles and capacity of inducing disease in the host [1]. Indeed, several organs and tissues have been described as possible targets of Bc infection; however, to date, hepatic tropism has never been convincingly reported. Here, we present the second definite case of hepatic abscess due to Bc in an immunocompetent host, the first to be managed and cured by percutaneous drainage and antibiotic treatment.

## 2. Case Report

**Figure 1:**
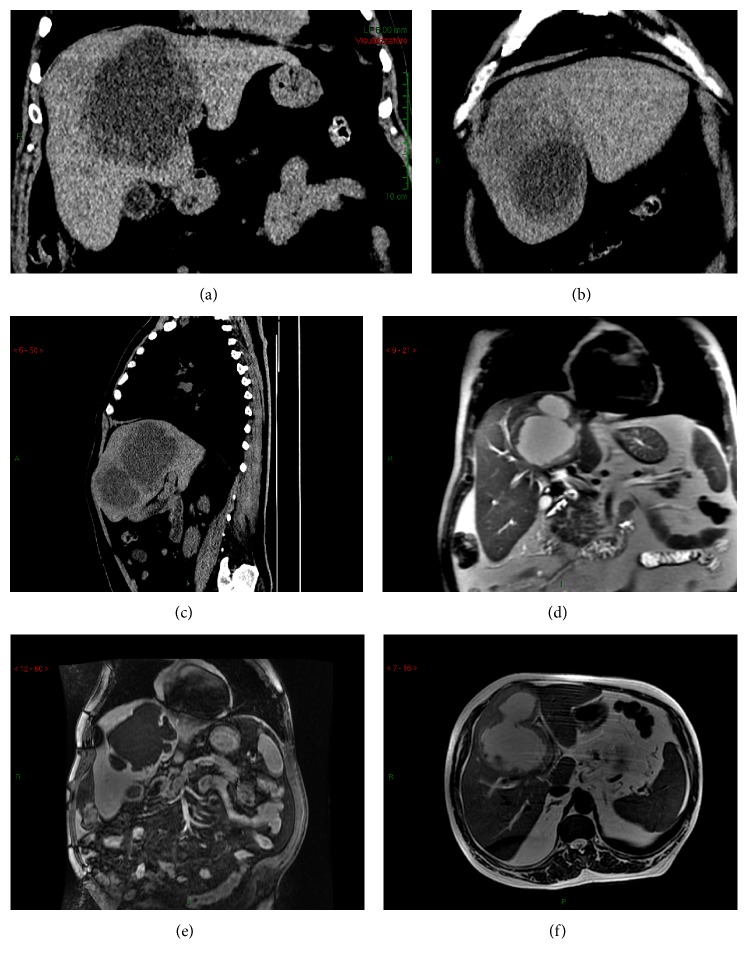
CT scan and MRI imaging of the liver lesions. (a) CT scan. Coronal plane. Evidence of the wider abscess as hypodense area with blurred edges. It occupies the IV-V-VIII hepatic segments and its size is approximately 12 × 10 × 10 cm. (b) CT scan. Sagittal plane. Evidence of both abscesses into the right hepatic lobe. (c) CT scan. Coronal plane. Evidence of the smaller abscess as hypodense area. It is located between the IV-V segments and its size is approximately 3 × 3 × 3 cm. (d) T2-weighted MRI. Coronal plane. Evidence of the wider abscess which determines ab estrinseco compression on the hepatic hilum. Moderate dilation of bile ducts of the VIII segment is also visible. (e) T2-weighted MRI. Axial plane. Evidence of communication between the two abscesses. (f) T1-weighted MRI after contrast medium. Coronal plane. Evidence of the wider abscess surrounded by communicating satellite lesions.

A 59-year-old man was admitted to our hospital complaining of fatigue, nausea, right upper quadrant heaviness, and dyspepsia, with an approximately 10 Kg weight loss in the previous 20 days. At admission, he was apyretic and he denied fever and other gastrointestinal symptoms in the previous weeks. On physical examination, an enlarged liver with a palpable round mass over the right hepatic lobe was detected. Laboratory tests showed mild leukocytosis (WBC: 10.130/*μ*L; neutrophils: 7.910/*μ*L), elevation of inflammatory indices (ESR: 110 mm/hour; CRP: 96 mg/dL), cholestasis with mild hyperbilirubinemia (alkaline phosphatase: 1767 U/L; total bilirubin: 1.1 mg/dL; direct bilirubin: 0.6 mg/dL), and normal transaminase, lipase, and creatinine levels. Blood cultures were not performed, since the patient was permanently apyretic. Abdominal computed tomography (CT) scan evidenced two formations (~12 cm and ~3 cm of maximal diameter) with a dense fluid internal content, occupying almost completely hepatic segments IV, V, and VIII (Figures 1(a)–1(c)). Abdominal ultrasound and magnetic resonance imaging (MRI) confirmed the lesions to have a solid thick wall and a fluid central nucleus, strongly suggesting their abscessual nature (Figures 1(d)–1(f)).

Both formations were percutaneously drained, with aspiration of ~1.7 liters of purulent fluid, which was sent for culture. While awaiting microbiological results, broad-spectrum empiric antimicrobial therapy was started with vancomycin (2 g/24 h), metronidazole (1.5 g/24 h), and amikacin (1.2 g/24 h). Culture resulted to be positive for Bc infection, and antimicrobial therapy with only vancomycin was therefore maintained. Anamnestic deepening was therefore carried out but did not reveal any suspicious information about the possible source of infection: in particular, the patient worked as an informatic technician, and he had not any recent or past history of intravenous drug abuse; moreover, he was not carrier of prosthetic heart valve or pacemaker/implantable cardioverter defibrillator or of in-dwelling central venous catheter. The patient denied previous surgery and recent dental procedures. As far as we know, 100% of Bc isolates are susceptible to vancomycin, and vancomycin is considered optimal for empirical treatment of Bc blood stream infections [2]. Clinical conditions and blood tests progressively improved, and, 5 days after abscess drainage, the patient was discharged with the indication to continue antibiotic treatment with levofloxacin 500 mg/daily for 15 additional days. Indeed, levofloxacin demonstrated excellent antimicrobial activity against Bc [2], and it was therefore chosen for the oral antibiotic treatment as outpatient. An ultrasonography performed one month after percutaneous drainage and the beginning of antibiotic treatment confirmed complete resolution of both abscesses.

## 3. Discussion

To the best of our knowledge, only one case of liver abscess due to Bc in an adult immunocompetent host has been previously reported [3]. However, in that case, the abscess underwent spontaneous rupture and the patient developed purulent peritonitis, dying five days after hospital admission, before the microorganism could have been identified and treated. In the setting of immunosuppression, two previous cases of multiple liver abscesses caused by Bc have been reported in two patients affected by acute leukemia [4, 5]. To note, in these two cases, Bc was isolated from blood culture and not directly from abscessual fluid, and also these two patients presented a very poor prognosis, dying five days and five months after diagnosis, respectively [4, 5].

Bc is mainly responsible for two foodborne illnesses, that is, the diarrheal and emetic (vomiting) syndrome [6]. However, Bc is recognized as a pathogen for not only the GI tract, and the spectrum of infections includes fulminant bacteremia, central nervous system infections, endophthalmitis, osteomyelitis, urinary tract infections, cutaneous infections, endocarditis, and pneumonia [6]. This is probably due to the several exoenzymes (hemolysins, phospholipases, cytotoxin K, etc.) which Bc is able to produce in addition to emesis-inducing toxin and the three pore-forming enterotoxins [6]. According to our case and to this brief review of the pertinent literature, the liver should be considered as another possible target of Bc, more frequently in the immunocompromised but possibly also in the immunocompetent host. Hepatic involvement produces abscessual lesions and is possibly associated with fatal evolution; however, if promptly recognized, Bc liver abscesses can be definitely cured by percutaneous drainage and antibiotic treatment. In our case, we did not identify possible risk factors for Bc infection. Notwithstanding the fact that blood cultures were not performed, the lack of fever and of any other possible clinical manifestations of bacteremia argues against the spread of the microorganism into the bloodstream. In contrast with the previous mentioned cases [4, 5], the competence of the immune system likely contributed to the good prognosis.
